# Development of an in vitro diagnostic method to determine the genotypic sex of *Xenopus laevis*

**DOI:** 10.7717/peerj.6886

**Published:** 2019-05-01

**Authors:** Amin Eimanifar, John Aufderheide, Suzanne Z. Schneider, Henry Krueger, Sean Gallagher

**Affiliations:** Aquatic Department, Performing Laboratory, Eurofins EAG Agroscience, LLC, Easton, MD, USA

**Keywords:** Genetic sex determination, *Xenopus laevis*, *Taq*Man® real-time PCR, OCSPP-LAGDA

## Abstract

A genotypic sex determination assay provides accurate gender information of individuals with well-developed phenotypic characters as well as those with poorly developed or absent of phenotypic characters. Determination of genetic sex for *Xenopus laevis* can be used to validate the outcomes of Tier 2 amphibian assays, and is a requirement for conducting the larval amphibian growth and development assay (LAGDA), in the endocrine disruptor screening program (EDSP), test guidelines. The assay we developed uses a dual-labeled *Taq*Man probe-based real-time polymerase chain reaction (real-time PCR) method to determine the genotypic sex. The reliability of the assay was tested on 37 adult specimens of *X. laevis* collected from in-house cultures in Eurofins EAG Agroscience, Easton. The newly designed *X. laevis*-specific primer pair and probe targets the DM domain gene linked-chromosome W as a master female-determining gene. Accuracy of the molecular method was assessed by comparing with phenotypic sex, determined by necropsy and histological examination of gonads for all examined specimens. Genotypic sex assignments were strongly concordant with observed phenotypic sex, confirming that the 19 specimens were male and 18 were female. The results indicate that the *Taq*Man® assay could be practically used to determine the genetic sex of animals with poorly developed or no phenotypic sex characteristics with 100% precision. Therefore, the *Taq*Man® assay is confirmed as an efficient and feasible method, providing a diagnostic molecular sex determination approach to be used in the amphibian endocrine disrupting screening programs conducted by regulatory industries. The strength of an EDSP is dependent on a reliable method to determine genetic sex in order to identify reversals of phenotypic sex in animals exposed to endocrine active compounds.

## Introduction

The African clawed frog (*Xenopus laevis*) belongs to the Pipidae family of frogs, all of which have an aquatic lifestyle in adulthood (Franco et al., 2001). *Xenopus laevis* is representative of an important group of amphibians that have been widely used for biomedical and environmental research and have a fully mapped genome (Flament, 2016; Session et al., 2016). African clawed frogs (*X. laevis*) offer a promising model to investigate the effect of potential endocrine disrupting chemicals (EDCs) within the framework of the United States Environmental Protection Agency’s (EPA’s), endocrine disruptor screening program (EDSP), larval amphibian growth and development assay (LAGDA) (United States Environmental Protection Agency (US EPA), 2015). *Xenopus laevis* has also been used as a model to study sex chromosome evolution (Furman & Evans, 2016). Sex determination in *X. laevis* follows a ZW gametic system, where females are heterogametic (ZW) and males are homogametic (ZZ). *Xenopus laevis* is a sexually monomorphic species and the adult females are larger than males (Babošová et al., 2018). Identification of phenotypic sex reversals in sexually mature frogs is not possible without a reliable genetic sex assay. In *X. laevis*, a gene called DM domain gene linked-W chromosome (DM-W) is a master female sex determination gene (Yoshimoto et al., 2008). The DM-W gene appeared in an ancestor of *X. laevis* after divergence from the ancestor of *X. tropicalis*, and is present in many close relatives of *X. laevis* (Bewick, Anderson & Evans, 2011).

In theory, a standard PCR amplification should generate two bands in females and a single band in males (United States Environmental Protection Agency (US EPA), 2015). Applications of traditional gel electrophoresis methods are labor-intensive and have increased variability in electrophoresis resolution power, thus limiting the precision of sex determination in the target specimens.

Molecular markers have been useful tools to identify the genotypic sex of individuals when the sex is difficult or impossible to determine due to the lack of phenotypic characters (Von Bargen, Smith & Rueth, 2015). Applications of molecular-based approaches for sex determination have become a more significant assay especially when a species shows no sexual dimorphism in an organism which prohibits scientists to accurately determine an external sex. Additionally, these approaches allow sex determination prior to the development of phenotypic characters in those species with clear sexual dimorphism, enabling scientists to determine the sex of juveniles. These methods could also be used in behavioral and conservation studies (Morinha et al., 2011).

To overcome the current limitation of traditional methods, new high-throughput methods such as capillary electrophoresis and real-time PCR using *Taq*Man® probes have been proposed as a better technique to sex specimens (Lee et al., 2010; Von Bargen, Smith & Rueth, 2015). These methods are sensitive, species-specific, efficient and cost-effective compared with traditional methods for collecting genotypic data (Morinha et al., 2011). Real-time PCR with *Taq*Man® chemistry method has been frequently used to determine the gender of birds (Chang et al., 2008), fish (Flynn et al., 2010; Von Bargen, Smith & Rueth, 2015) and pigs (Abdulmawjood et al., 2012).

The goal of the present study was 2-fold. The first was to develop a *Taq*Man® chemistry detection approach by modifying a method presented in the EPA guideline, based on amplification of DM-W gene to determine the genetic sex of *X. laevis* specimens. This assay is more sensitive and cost-efficient than the assay currently described in the EDSP test guideline and provides an alternate approach to genetic sex identification. The second goal was to confirm the reliability of the new assay by histological examination of the gonads of each specimen (oviduct and testis) as secondary sex characteristic according to the procedures described in the EDSP test guideline. The use of sexually mature frogs provided fully developed gonads for histological sex identification.

## Materials and methods

### Frog sources

Frogs used for genotyping were obtained from the in-house culture facility at the Performing Laboratory, Eurofins EAG Agroscience in Easton, MD, USA, and had exceeded their effective age (∼36 months) for use in other laboratory research. Sexually mature frogs were purchased from two commercial suppliers Xenopus Express Inc. and Xenopus I. The frogs were maintained under optimum laboratory conditions including temperature (22 ± 2 °C), dissolved oxygen (above 80% mg/L), pH ∼8.0, photoperiod 16 h light/8 h dark (30 min. transition period) and fed a commercially available diet three times per week. A toe clip of each adult animal was sampled for genetic analysis, followed by dissection and removal and preservation of the gonads for histological evaluation. An accurate genetic sex determination method for *X. laevis* is a requirement for testing within the framework of the United States EPA’s EDSP, and for meeting similar OECD guideline requirements. The use of these animals for development of real-time PCR genetic sex determination assay not only advanced the science in this area, but also ensured that the amount of useful data derived from these animals was maximized, eliminating the need for novel test animals.

### DNA extraction

Test animals were anesthetized by immersion in tricaine methanesulfonate (1 gr/L) (MS 222; Sigma-Aldrich, St. Louis, MI, USA), decapitated and dissected with sterilized sharp forceps. Toe clips from each animal were dissected by scalpel from one hind limb, placed in ethyl alcohol (Sigma-Aldrich, St. Louis, MI, USA) and stored at −80 °C for later DNA extraction. DNA extraction was performed by placing toe clip samples from each frog in 20 µL of proteinase K (2 mg/mL stock solution) and incubated at 60 °C for 35 min. All DNA extractions were followed using the Qiagen DNeasy® Blood and Tissue extraction kit (Qiagen, Germantown, MD, USA, Cat. No. 69506), eluted in 200 µL AE buffer and stored at −20 °C until genetic analyses were performed.

### Genotypic sex determination analysis

#### Assay design

For sex determination, a 260-bp fragment of the female-specific DM-W was targeted with primers DM-W Forward 5′-GGGCAGAGTCACATATACTG-3′; DM-W Reverse 5′-CCACACCCAGCTCATGTAAAG-3′ (Yoshimoto et al., 2008) and a newly designed dual-labeled fluorescent probe using *Taq*Man® chemistry detection method (5′-/56-FAM/AGCATACCCGATGAACAAAGAACAAGC/3BHQ_1/-3′). Primers and probe sequences were designed from *X. laevis* complete DNA sequence (GenBank accession number: AP013026.1) using PrimerQuest tool as implemented in the Integrated DNA Technologies (Owczarzy et al., 2008).

### Real-time PCR

Each PCR reaction contained 12.5 µL Rotor-Gene Multiplex PCR Kit (Qiagen, Germantown, MD, USA, Cat. No. 204774), 0.62 µM each primer, 0.14 µM probe, DNA template 4 µL, 0.24 µM 18S rRNA and nuclease-free water to a final volume of 25 µL in Rotor-Gene Q 2 plex 72-well reaction plates (Qiagen, Germantown, MD, USA). Each sample was analyzed in duplicate for molecular sexing and both DM-W and 18S rRNA were multiplexed in duplex PCRs. We used 18S rRNA as positive internal control primer in each sample to minimize the experimental errors (Banaras et al., 2012). The cycling conditions were 50 °C for 2 min, 95 °C for 5 min, followed by 30 cycles of 95 °C for 40 s and 60 °C for 1 min, respectively. The quality and precision of amplification curves for each sample were carefully checked to ensure no pseudo amplification occurred.

### Phenotypic sex based on gonad histology

Gonads of each specimen including testes and oviducts were carefully dissected, separated from adjacent tissue, fixed in Davidson’s solution (24 h) and subsequently rinsed in 70% ethanol for 30 min and placed in 10% formalin until histological evaluation for phenotypic sex identification. Phenotypic sex of each animal was also identified based on gross gonadal anatomy during dissection.

Histological sections were prepared for all specimens without prior information on genetic sex. Histopathological evaluation was performed according to the established protocol as implemented by United States, EPA EDSP, LAGDA guideline (United States Environmental Protection Agency (US EPA), 2015). Gonads were embedded in paraffin with a Sakura Tissue-Tek TEC station, sectioned on a Microm HM 325 microtome into five µm thickness, stained with hematoxylin and eosin (HE), and examined using brightfield microscopy. Images were taken with a Sony A7R II digital camera. Histological evaluation of gonads for phenotypic sex identification was performed by a board-certified pathologist with extensive experience in fish and frog histology.

## Results and discussion

**Figure 1:**
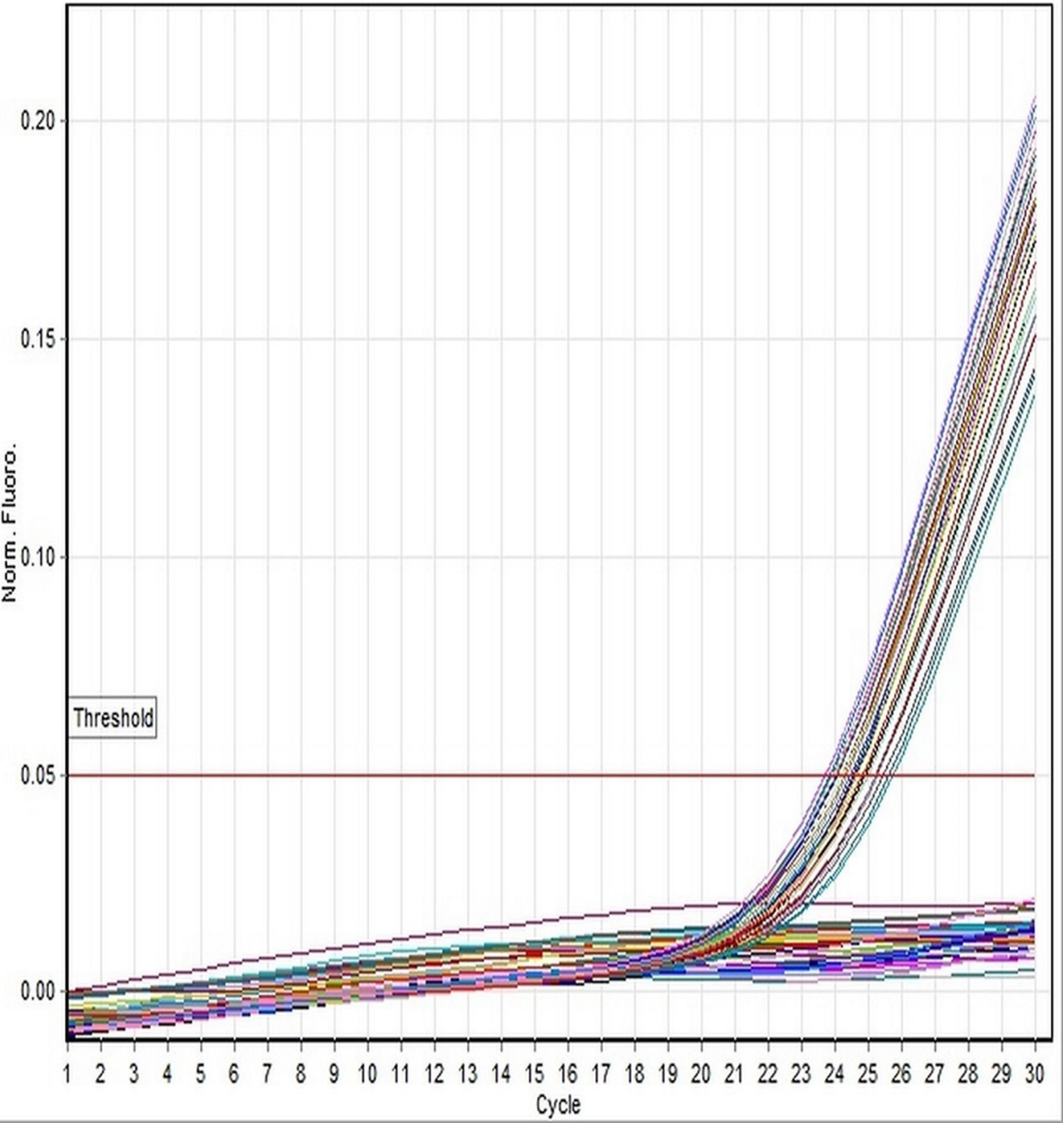
Linear amplification curves of DM-W gene for all specimens using the *Taq*Man-based assay. All amplification curves crosses the solid threshold line denoted female’s genotype and the curves occurred below the threshold line are male genotypes. All curves indicate the amplification of DM-W gene generated by Rotor-Gene Q Real-time PCR with optimum cycling conditions, followed by 30 cycles. The threshold line was set automatically by the software.

**Figure 2:**
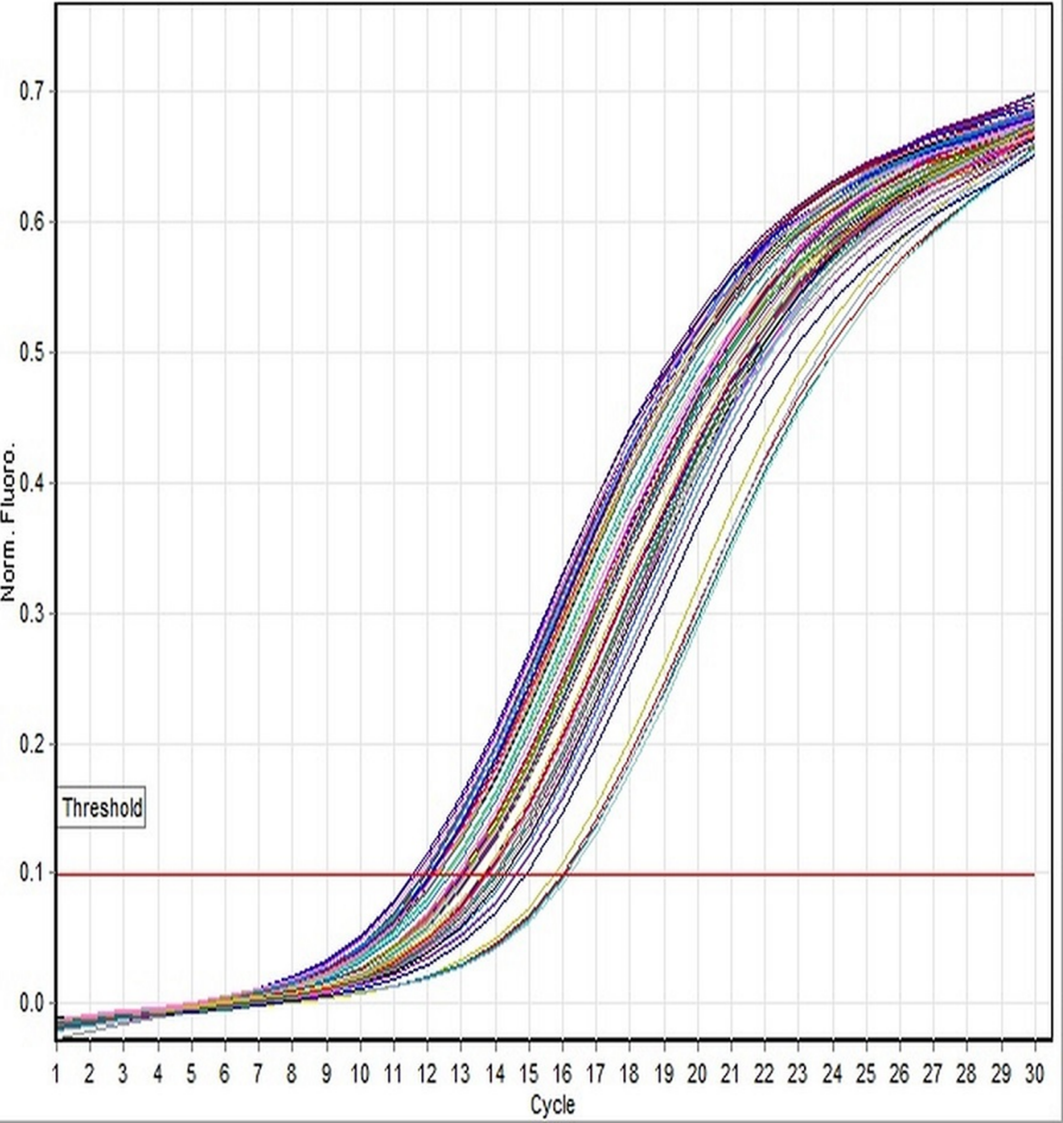
Linear amplification curves of 18S rRNA gene for all specimens using the *Taq*Man-based assay. All specimens showed stable expression curves occurred between 10 and 14 cycles. All curves indicate the amplification of 18S rRNA gene generated by Rotor-Gene Q Real-time PCR with optimum cycling conditions, followed by 30 cycles. The threshold line was set automatically by the software.

**Figure 3:**
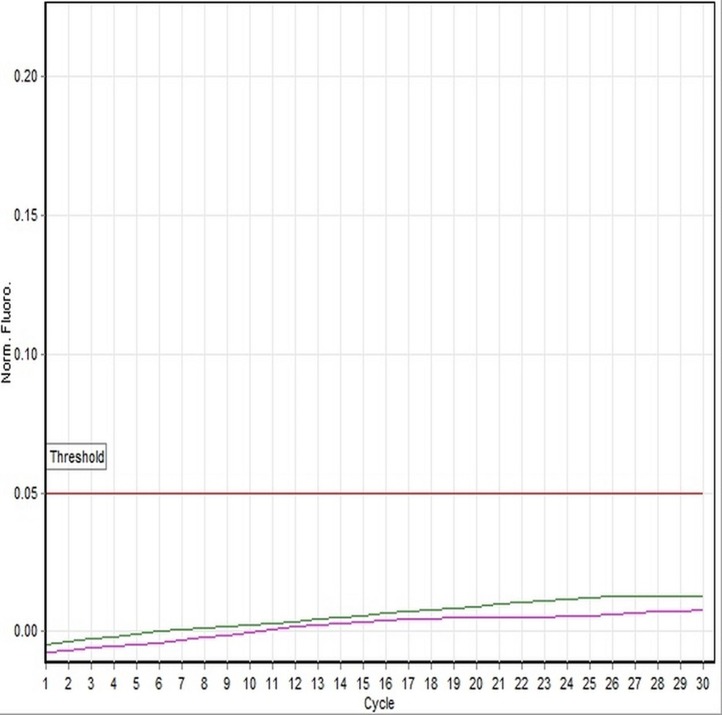
A representative example of linear amplification curve of a male specimen (100A-116-DNA-5) using the *Taq*Man-based assay. The plot indicates that the sample No. 5 did not generate a DM-W amplification curve by Rotor-Gene Q Real-time PCR, which occurred below the threshold line set by the software.

**Figure 4:**
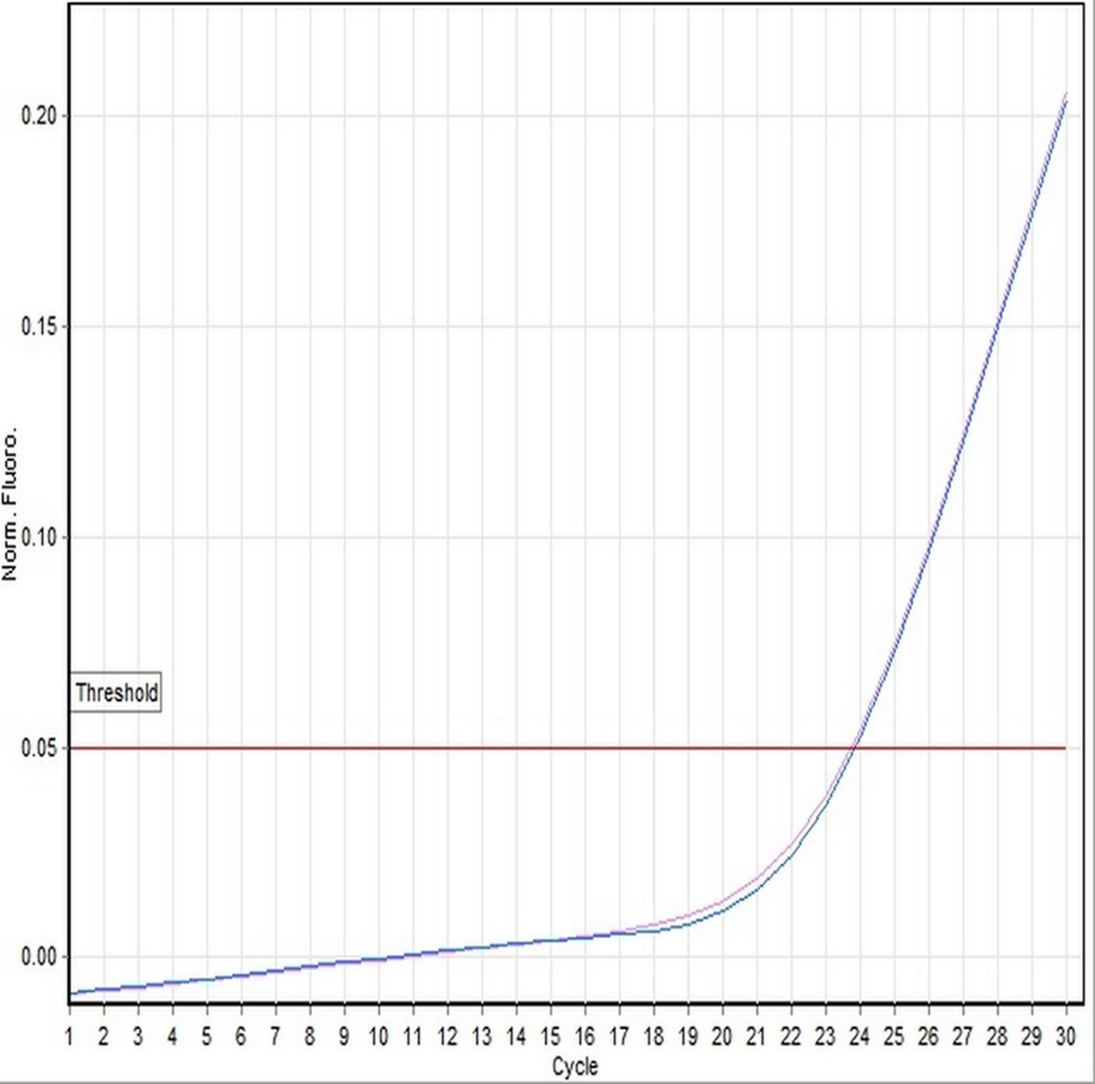
A representative example of linear amplification curve of a female specimen (100A-116-DNA-35) using the *Taq*Man-based assay. The plot indicates that the sample No. 35 did generate a DM-W amplification curve by Rotor-Gene Q Real-time PCR, which occurred above the threshold line set by the software.

In the present study, we have focused on genetic and histological sexing of 37 sexually matured adult *X. laevis* specimens. Real-time PCR analysis using DM-W *Taq*Man® system was conducted in order to determine the gender of *X. laevis* specimens. Specimens that showed DM-W PCR amplification in the duplicate analyses indicated a female genotype, whereas no band of PCR amplification of DM-W gene indicated a male genotype. Of the 37 specimens sampled, 19 were determined to be male and 18 were determined to be female using PCR analysis. The PCR product of DM-W amplified gene was 260 bp. Toe clip specimens collected from hind limb of *X. laevis* were characterized with clear amplification of the DM-W and 18S rRNA genes from genomic DNA templates. The amplification of the DM-W occurred between 21 and 23 cycles and amplification of 18S rRNA occurred between 10 and 13 cycles in all specimens (Figs. 1 and 2). Since DM-W and 18S rRNA PCR amplicons were detected in all female specimens, the precision and reliability of *Taq*Man® real-time PCR system was 100%. An example of DM-W amplification curve for male and female were shown in Figs. 3 and 4. The detailed output results of cycle of threshold (Ct) generated by Rotor-Gene Q for DM-W and 18S rRNA markers of each specimens are presented in Tables S1–S4. The applicability of *Taq*Man® real-time PCR system to determine the genetic sex of Japanese rice fish, medaka (*Oryzias latipes*) was confirmed by Flynn et al. (2010).

**Figure 5:**
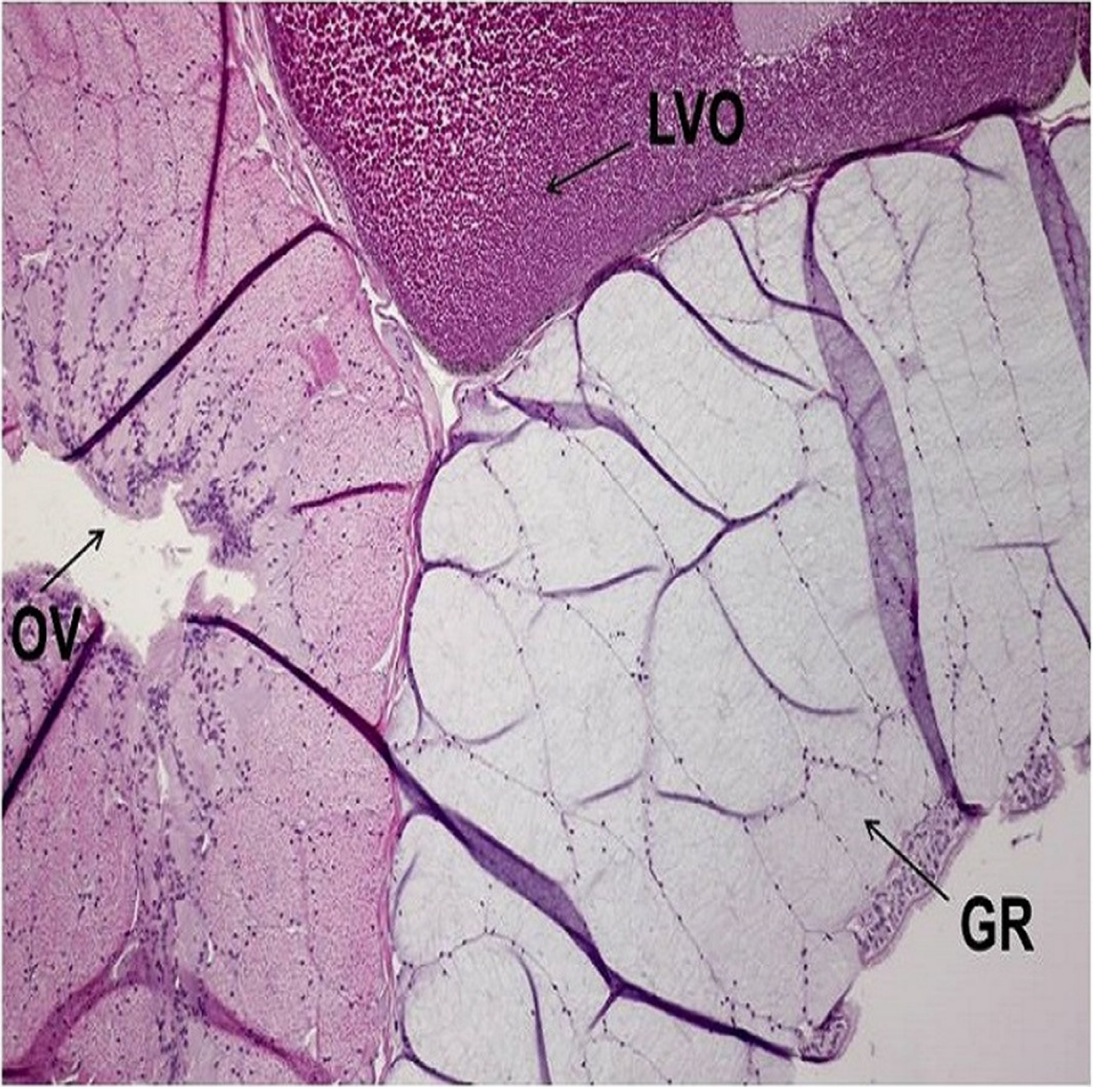
A representative of micrographic image of oviduct stained with Hematoxylin and eosin (HE), magnified at 10×. The characteristic structures for Oviduct (OV), Late Vitellogenic Oocyte (LVO) and Glandular Region (GR) are identified. The image produced by the histological sectioning of the specimen following the procedures published in OECD-LAGDA guideline, stained with Hematoxylin and eosin (HE). A female biological endpoint was confirmed by a certified pathologist.

**Figure 6:**
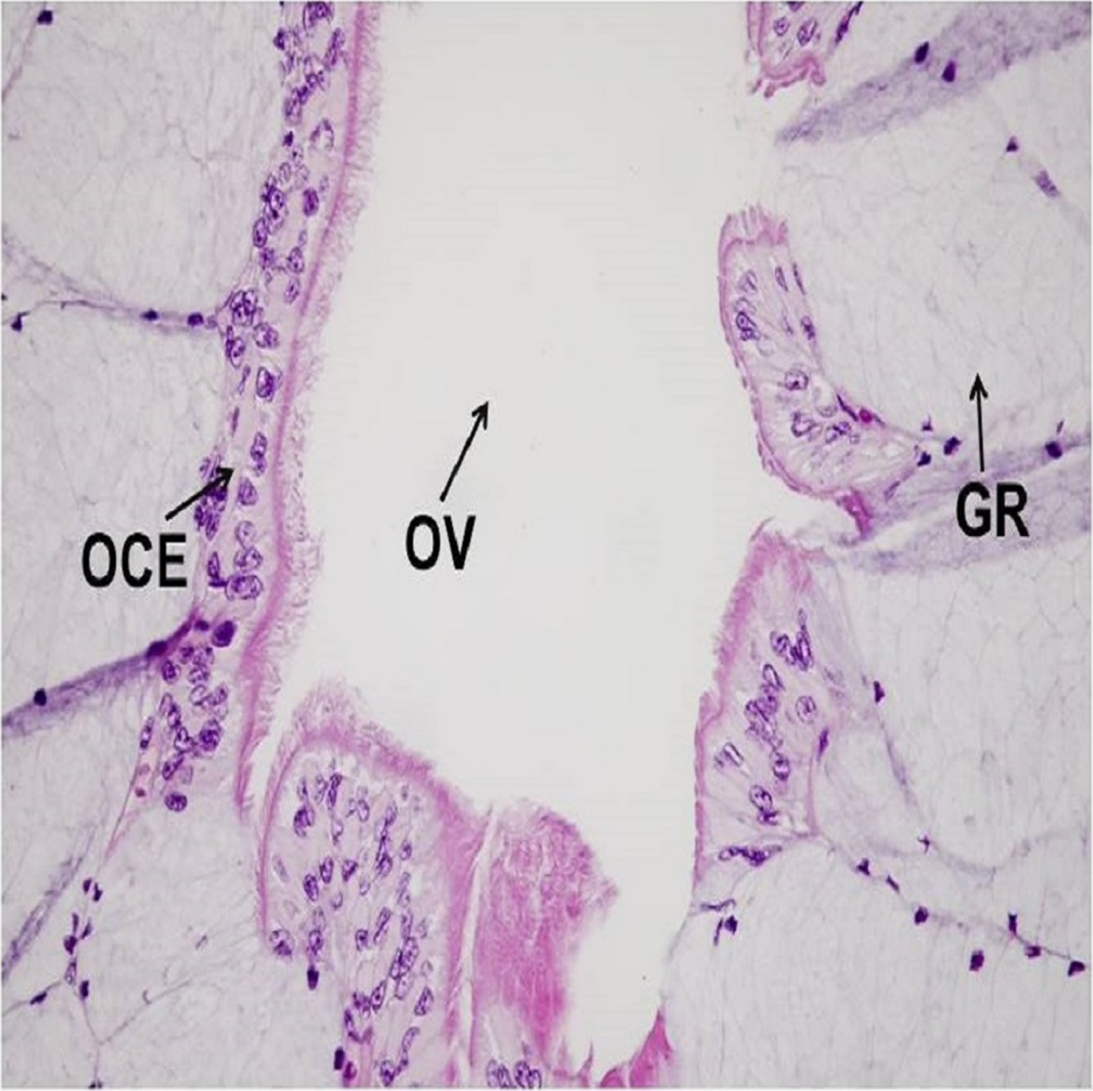
A representative of micrographic image of oviduct stained with Hematoxylin and eosin (HE), magnified at 40×. The characteristic structures for Oviduct (OV), Oviduct Ciliated Epithelium (OCE) and Glandular Region (GR) are identified. The image produced by the histological sectioning of the specimen following the procedures published in OECD-LAGDA guideline, stained with Hematoxylin and eosin (HE). A female biological endpoint was confirmed by a certified pathologist.

**Figure 7:**
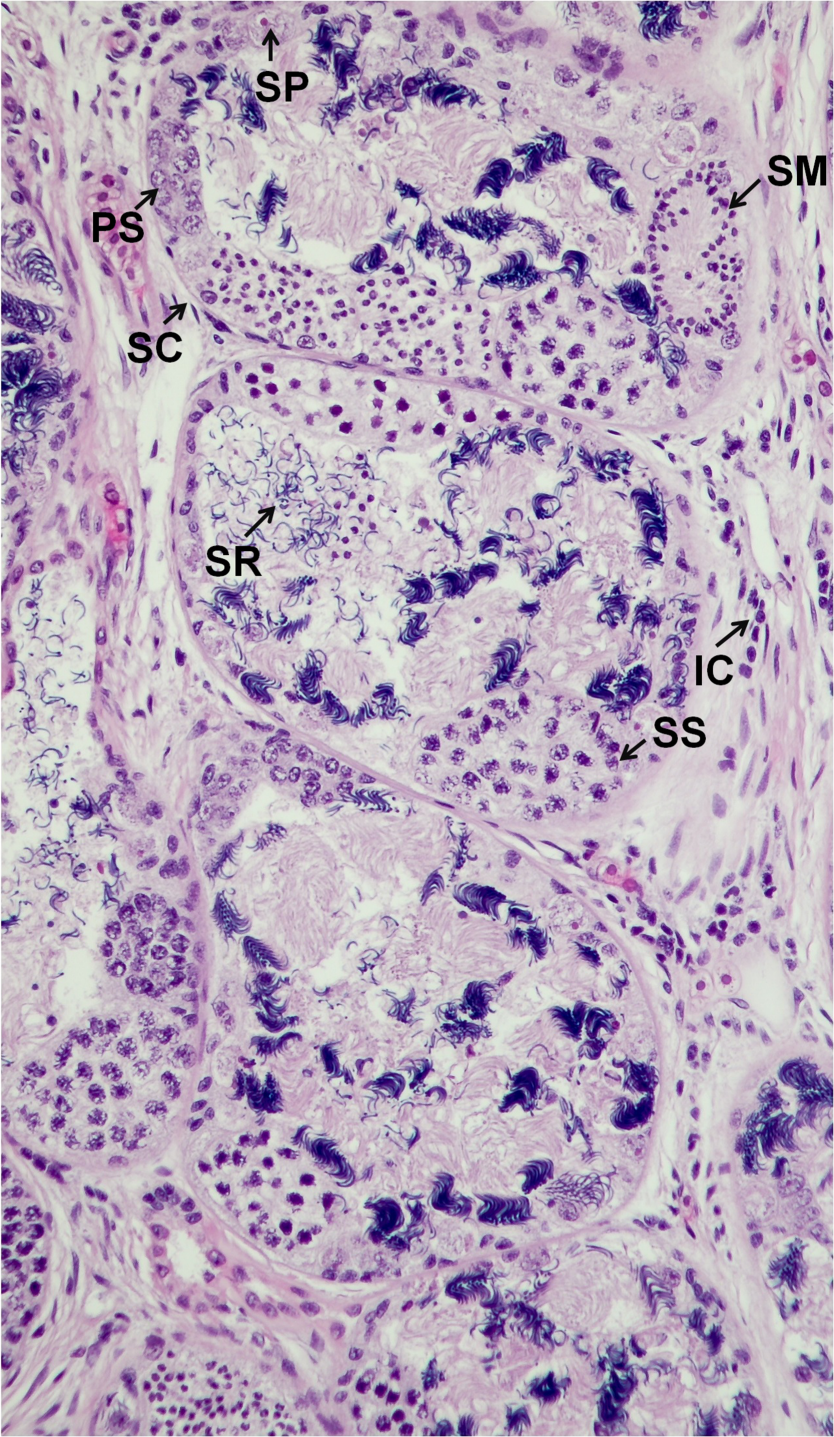
A representative of micrographic image of testis stained with Hematoxylin and eosin (HE), magnified at 20×. The characteristic structures for Sperm (SR), Spermatogonia (SP), Primary Spermatocytes (PS), Secondary Spermatocytes (SS), Spermatids (SM), Interstitial Cells (IC) and Sertoli Cell (SC) are identified. The image produced by the histological sectioning of the specimen following the procedures published in OECD-LAGDA guideline, stained with Hematoxylin and eosin (HE). A male biological endpoint was confirmed by a certified pathologist.

**Figure 8:**
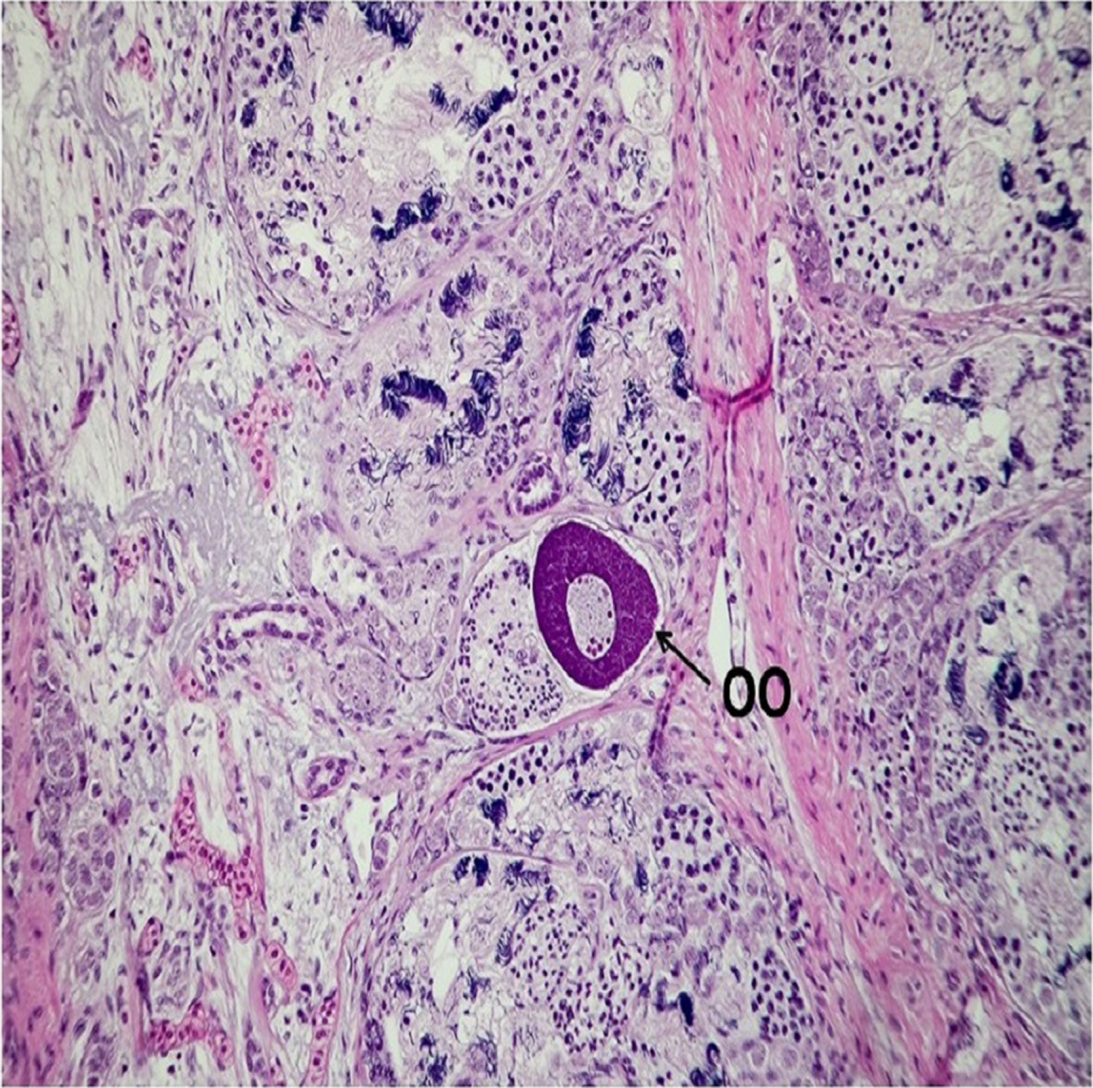
Micrographic image of a male testis with presence of oocyte inside seminiferous tubules, stained with Hematoxylin and eosin (HE), magnified at 20×. The characteristic structure for Oocyte (OO) is identified. The image produced by the histological sectioning of the specimen following the procedures published in OECD-LAGDA guideline, stained with Hematoxylin and eosin (HE). The presence of an oocyte inside seminiferous tubules is called “testis-ovum”.

Histological sections from each specimen were screened to determine histological sex as a confirmatory method. This method is typically used to determine the gonad phenotype of fish after exposure to putative EDCs in regulatory testing laboratories (Flynn, Swintek & Johnson, 2013). Oviducts are female secondary sex characteristics that function in oocyte maturation during reproduction (Wake & Dickie, 1998). Oviducts were identified by their epithelial cell lining (1–3 cell layers), which is comparable in size to the Wolffian duct. Testes were recognized by individual spermatogonia, spermatocytes and/or seminiferous cords or tubules (United States Environmental Protection Agency (US EPA), 2015). There were no gross morphological abnormalities observed in the gonads of all specimens. Gonad histological sex confirmed the genotypes of all specimens, indicative of the robustness and precision of the newly developed real-time PCR assay for sex determination of *X. laevis* specimens. The concatenated results from genetic and histological sexing are shown in Table 1. A summary of histology results reported by a pathologist is provided in Table S5. All specimen transmittal forms including specimen processing instruction, histology processing log, HE staining, specimen transmittal of slides, blocks and preserved remnants to temporary storage are provided in Table S6. A representative image for oviduct is presented in Figs. 5 and 6. A histology image of testis is provided in Fig. 7; Figs. S1 and S2. Of interest was one male frog confirmed by both histology and genetic sex that had the presence of an oocyte inside seminiferous tubules (Fig. 8). While the exact reason for the abnormal finding which is called “testis-ovum” in this one frog was unknown, it demonstrates that having a good method for determining genetic sex helps to understand the extent of phenotypic changes and the possibility of sex reversal. The current phenomenon was not reported for Xenopus, but was known from Rhacophorus and Rana (Pelophylax). They were described in more than 50% of males *Rana nigromaculata* studied by Iwasawa & Kobayashi (1976), and Kobayashi & Iwasawa (1988). Differentiation of female germ cells inside testes might be caused by dysfunction of hormonal control, i.e. low androgen synthesis or insufficient level of receptors. Testis-ova were also observed in *Rhacophorus arboreus* (Tanimura & Iwasawa, 1989), *Rana catesbeiana* (Hsu, Chiang & Liang, 1973; Hsu & Wang, 1981), and occasionally in *Rana lessonae* and *Rana esculenta* (Ogielska & Bartmańska, 1999). It is possible to completely reverse the phenotypic sex of *X. laevis* with known endocrine active substances such as 17β-estradiol (Oka et al., 2006). Having the genetic sex helps determine if complete sex reversal may have occurred.

**Table 1:** Concatenated results of genotypic and histology sexes for all *Xenopus laevis* studied specimens.

No.	Specimens	DM-W*	Genotypic sex	Histology sex
1	100A-116-DNA-1	−	Male	Male
2	100A-116-DNA-2	−	Male	Male
3	100A-116-DNA-3	−	Male	Male
4	100A-116-DNA-4	−	Male	Male
5	100A-116-DNA-5	−	Male	Male
6	100A-116-DNA-6	−	Male	Male
7	100A-116-DNA-7	−	Male	Male
8	100A-116-DNA-8	−	Male	Male
9	100A-116-DNA-9	−	Male	Male
10	100A-116-DNA-10	−	Male	Male
11	100A-116-DNA-11	−	Male	Male
12	100A-116-DNA-12	−	Male	Male
13	100A-116-DNA-13	−	Male	Male
14	100A-116-DNA-14	−	Male	Male
15	100A-116-DNA-15	−	Male	Male
16	100A-116-DNA-16	−	Male	Male
17	100A-116-DNA-17	−	Male	Male
18	100A-116-DNA-18	−	Male	Male
19	100A-116-DNA-19	−	Male	Male
20	100A-116-DNA-20	+	Female	Female
21	100A-116-DNA-21	+	Female	Female
22	100A-116-DNA-22	+	Female	Female
23	100A-116-DNA-23	+	Female	Female
24	100A-116-DNA-24	+	Female	Female
25	100A-116-DNA-25	+	Female	Female
26	100A-116-DNA-26	+	Female	Female
27	100A-116-DNA-27	+	Female	Female
28	100A-116-DNA-28	+	Female	Female
29	100A-116-DNA-29	+	Female	Female
30	100A-116-DNA-30	+	Female	Female
31	100A-116-DNA-31	+	Female	Female
32	100A-116-DNA-32	+	Female	Female
33	100A-116-DNA-33	+	Female	Female
34	100A-116-DNA-34	+	Female	Female
35	100A-116-DNA-35	+	Female	Female
36	100A-116-DNA-36	+	Female	Female
37	100A-116-DNA-37	+	Female	Female

**Notes:**

Each data point indicates the genetic identity of each specimen confirmed by genetic and histology approaches.

* Detection of DM-W gene by real-time PCR. +: positive, −: negative.

## Conclusions

This study has demonstrated the reliability and usefulness of the *Taq*Man® real-time PCR method for determining the genetic sex of *X. laevis* from toe clip specimens. These are functional assays that only require a small quantity of total DNA template without purification of nucleic acid. The assay is useful when used in conjunction with animal phenotypic characters and gonad morphology to confirm sex and to identify potential sex reversal in *X. laevis*.

The real-time amplification curves provide a unique opportunity to distinguish amplicon derived from DM-W heterozygous sex chromosomes from female specimens.

The *Taq*Man® real-time PCR assay offers a reliable, precise, time-efficient and cost-effective method to monitor phenotypic sex changes of *X. laevis* in the EDSP, OCSPP 890.2300 LAGDA at the regulatory agencies and in other research with *X. laevis*.

## Supplemental Information

10.7717/peerj.6886/supp-1 Supplemental Information 1 The detailed DM-W gene Ct values for specimen Nos. 1−36 using *Taq*Man-based assay. Specimens that did not generate a Ct value denote a male genotype and specimens that generated a Ct value denote a female genotype. Each specimen was analyzed in duplicate, confirmed by the presence or absence of DM-W gene amplification generated by Rotor-Gene Q real-time PCR with optimum cycling conditions, followed by 30 cycles. The Ct value was used as an endpoint to determine the genetic identity of specimen Nos. 1–36.

10.7717/peerj.6886/supp-2 Supplemental Information 2 The detailed DM-W gene Ct value for specimen No. 37 using *Taq*Man-based assay. A specimen that generated a Ct value denotes a female genotype. A single specimen No. 37 was analyzed in duplicate, confirmed by the presence of DM-W gene amplification generated by Rotor-Gene Q real-time PCR with optimum cycling conditions, followed by 30 cycles. The Ct value was used as an endpoint to determine the genetic identity of specimen No. 37.

10.7717/peerj.6886/supp-3 Supplemental Information 3 The detailed 18S rRNA gene amplification Ct values for specimen Nos. 1−36 using *Taq*Man-based assay. All specimens generated a Ct value regardless of sex. Each specimen was analyzed in duplicate, produced Ct value for the amplification of 18S rRNA as a reference housekeeping marker. All values generated by Rotor-Gene Q real-time PCR with optimum cycling conditions, followed by 30 cycles.

10.7717/peerj.6886/supp-4 Supplemental Information 4 The detailed 18S rRNA gene amplification Ct value for specimen No. 37 using *Taq*Man-based assay. A specimen generated a Ct value regardless of sex. A single specimen No. 37 was analyzed in duplicate, produced Ct value for the amplification of 18S rRNA as a reference housekeeping marker. The values generated by Rotor-Gene Q real-time PCR with optimum cycling conditions, followed by 30 cycles.

10.7717/peerj.6886/supp-5 Supplemental Information 5 A summary of histology sex evaluation for all specimens. The data indicate the evaluation of histological sex of all specimens approved by a certified pathologist.

10.7717/peerj.6886/supp-6 Supplemental Information 6 A transmittal form containing specimen processing instructions, histology processing log, HE staining, specimen transmittal of slides, blocks and preserved remnants to temporary storage. Each form was generated based on GLP guidelines and was used to record the data during histology sectioning by an experienced technician.

10.7717/peerj.6886/supp-7 Supplemental Information 7 A representative of micrographic image of testis for specimen No. 5 stained with HE. The image produced by the histological sectioning of the specimen No. 5 following the procedures published in OECD-LAGDA guideline, stained with HE. A male biological endpoint was confirmed by a certified pathologist.

10.7717/peerj.6886/supp-8 Supplemental Information 8 A representative of micrographic image of testis for specimen No. 2 stained with HE. The image produced by the histological sectioning of the specimen No. 2 following the procedures published in OECD-LAGDA guideline, stained with HE). A male biological endpoint was confirmed by a certified pathologist.

## Additional Information and Declarations

### Data Availability

The following information was supplied regarding data availability: The raw data are available in the Supplementary Files (Figs. S1 and S2; Tables S1–S6).
