# Supplementary material for: Development of an in vitro diagnostic method to determine the genotypic sex of Xenopus laevis
Source: PeerJ. 2019 May 1;7:e6886. doi: 10.7717/peerj.6886 (PMC6500372; doi:10.7717/peerj.6886)
Supplement: Supplemental Information 1 — Each specimen was analyzed in duplicate, confirmed by the presence or absence of DM-W gene amplification generated by Rotor-Gene Q real-time PCR with optimum cycling conditions, followed by 30 cycles. The Ct value was used as an endpoint to determine the genetic identity of specimen Nos. 1–36. [file peerj-07-6886-s001.doc]

| 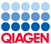 | [www.qiagen.com](http://www.qiagen.com/) |
| --- | --- |

# Quantitation Report

#### Experiment Information

| Run Name | 100A-116_DMW_Assay 1 |
| --- | --- |
| Run Start | 8/21/2018 2:22:52 PM |
| Run Finish | 8/21/2018 3:47:25 PM |
| Operator | Amin Eimanifar |
| Notes |  |
| Run On Software Version | Rotor-Gene Q Software 2.3.1.49 |
| Run Signature | The Run Signature is valid. |
| Gain Green | 5. |
| Gain Yellow | 5. |
| Machine Serial No. | 0713167 |

#### Quantitation Information

| Threshold | 0.050 |
| --- | --- |
| Left Threshold | 1.000 |
| Standard Curve Imported | No |
| Standard Curve (1) | N/A |
| Standard Curve (2) | N/A |
| Start normalising from cycle | 1 |
| Noise Slope Correction | No |
| No Template Control Threshold | % 0 |
| Reaction Efficiency Threshold | Disabled |
| Normalisation Method | Dynamic Tube Normalisation |
| Digital Filter | Light |
| Sample Page | Page 1 |
| Imported Analysis Settings |  |

#### Profile

| Cycle | Cycle Point |
| --- | --- |
| Hold 1 | Hold @ 50°C, 2min 0s |
| Hold 2 | Hold @ 95°C, 5min 0s |
| Cycling (30 repeats) | Step 1: Hold @ 95°C, 40s |
|  | Step 2: Hold @ 60°C, 60s, acquiring to Cycling A([Green][1][1],[Yellow][2][2]) |

#### Raw Data For Cycling A.Green


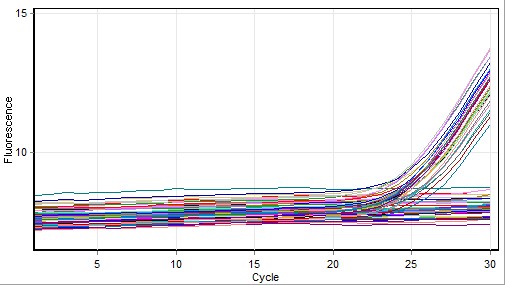


#### Quantitation data for Cycling A.Green


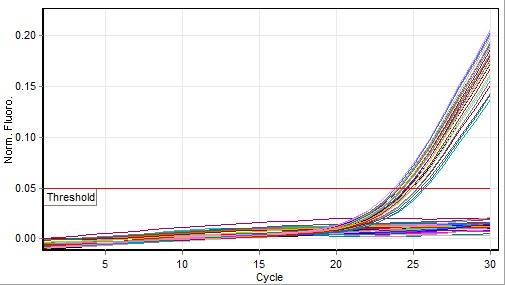


#### Standard Curve


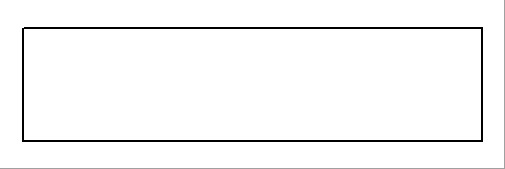


| No. | Color | Name | Type | Ct | Ct Comment | Given Conc (Copies) | Calc Conc (Copies) |
| --- | --- | --- | --- | --- | --- | --- | --- |
| 1 | 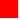 | 100A-116-DNA-1 | Unknown |  |  |  |  |
| 2 | 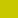 | 100A-116-DNA-1 | Unknown |  |  |  |  |
| 3 | 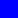 | 100A-116-DNA-2 | Unknown |  |  |  |  |
| 4 | 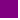 | 100A-116-DNA-2 | Unknown |  |  |  |  |
| 5 | 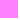 | 100A-116-DNA-3 | Unknown |  |  |  |  |
| 6 | 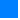 | 100A-116-DNA-3 | Unknown |  |  |  |  |
| 7 | 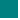 | 100A-116-DNA-4 | Unknown |  |  |  |  |
| 8 | 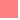 | 100A-116-DNA-4 | Unknown |  |  |  |  |
| 9 | 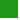 | 100A-116-DNA-5 | Unknown |  |  |  |  |
| 10 | 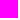 | 100A-116-DNA-5 | Unknown |  |  |  |  |
| 11 | 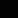 | 100A-116-DNA-6 | Unknown |  |  |  |  |
| 12 | 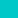 | 100A-116-DNA-6 | Unknown |  |  |  |  |
| 13 | 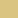 | 100A-116-DNA-7 | Unknown |  |  |  |  |
| 14 | 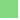 | 100A-116-DNA-7 | Unknown |  |  |  |  |
| 15 | 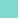 | 100A-116-DNA-8 | Unknown |  |  |  |  |
| 16 | 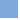 | 100A-116-DNA-8 | Unknown |  |  |  |  |
| 17 | 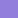 | 100A-116-DNA-9 | Unknown |  |  |  |  |
| 18 | 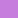 | 100A-116-DNA-9 | Unknown |  |  |  |  |
| 19 | 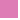 | 100A-116-DNA-10 | Unknown |  |  |  |  |
| 20 | 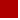 | 100A-116-DNA-10 | Unknown |  |  |  |  |
| 21 | 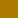 | 100A-116-DNA-11 | Unknown |  |  |  |  |
| 22 | 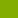 | 100A-116-DNA-11 | Unknown |  |  |  |  |
| 23 | 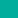 | 100A-116-DNA-12 | Unknown |  |  |  |  |
| 24 | 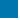 | 100A-116-DNA-12 | Unknown |  |  |  |  |
| 25 | 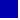 | 100A-116-DNA-13 | Unknown |  |  |  |  |
| 26 | 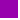 | 100A-116-DNA-13 | Unknown |  |  |  |  |
| 27 | 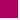 | 100A-116-DNA-14 | Unknown |  |  |  |  |
| 28 | 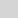 | 100A-116-DNA-14 | Unknown |  |  |  |  |
| 29 | 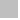 | 100A-116-DNA-15 | Unknown |  |  |  |  |
| 30 | 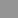 | 100A-116-DNA-15 | Unknown |  |  |  |  |
| 31 | 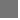 | 100A-116-DNA-16 | Unknown |  |  |  |  |
| 32 | 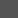 | 100A-116-DNA-16 | Unknown |  |  |  |  |
| 33 | 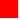 | 100A-116-DNA-17 | Unknown |  |  |  |  |
| 34 | 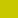 | 100A-116-DNA-17 | Unknown |  |  |  |  |
| 35 | 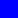 | 100A-116-DNA-18 | Unknown |  |  |  |  |
| 36 | 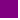 | 100A-116-DNA-18 | Unknown |  |  |  |  |
| 37 | 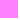 | 100A-116-DNA-19 | Unknown |  |  |  |  |
| 38 | 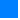 | 100A-116-DNA-19 | Unknown |  |  |  |  |
| 39 | 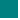 | 100A-116-DNA-20 | Unknown | 24.51 |  |  |  |
| 40 | 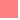 | 100A-116-DNA-20 | Unknown | 24.70 |  |  |  |
| 41 | 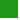 | 100A-116-DNA-21 | Unknown | 23.93 |  |  |  |
| 42 | 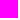 | 100A-116-DNA-21 | Unknown | 23.95 |  |  |  |
| 43 | 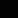 | 100A-116-DNA-22 | Unknown | 24.93 |  |  |  |
| 44 | 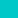 | 100A-116-DNA-22 | Unknown | 24.62 |  |  |  |
| 45 | 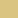 | 100A-116-DNA-23 | Unknown | 25.00 |  |  |  |
| 46 | 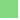 | 100A-116-DNA-23 | Unknown | 24.82 |  |  |  |
| 47 | 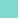 | 100A-116-DNA-24 | Unknown | 24.98 |  |  |  |
| 48 | 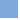 | 100A-116-DNA-24 | Unknown | 25.30 |  |  |  |
| 49 | 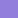 | 100A-116-DNA-25 | Unknown | 23.74 |  |  |  |
| 50 | 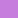 | 100A-116-DNA-25 | Unknown | 24.38 |  |  |  |
| 51 | 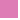 | 100A-116-DNA-26 | Unknown | 25.27 |  |  |  |
| 52 | 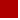 | 100A-116-DNA-26 | Unknown | 25.26 |  |  |  |
| 53 | 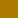 | 100A-116-DNA-27 | Unknown | 24.23 |  |  |  |
| 54 | 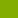 | 100A-116-DNA-27 | Unknown | 24.32 |  |  |  |
| 55 | 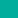 | 100A-116-DNA-28 | Unknown | 25.75 |  |  |  |
| 56 | 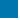 | 100A-116-DNA-28 | Unknown | 25.63 |  |  |  |
| 57 | 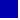 | 100A-116-DNA-29 | Unknown | 24.63 |  |  |  |
| 58 | 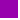 | 100A-116-DNA-29 | Unknown | 24.57 |  |  |  |
| 59 | 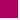 | 100A-116-DNA-30 | Unknown | 24.22 |  |  |  |
| 60 | 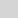 | 100A-116-DNA-30 | Unknown | 24.21 |  |  |  |
| 61 | 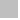 | 100A-116-DNA-31 | Unknown | 23.95 |  |  |  |
| 62 | 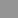 | 100A-116-DNA-31 | Unknown | 24.06 |  |  |  |
| 63 | 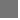 | 100A-116-DNA-32 | Unknown | 25.24 |  |  |  |
| 64 | 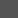 | 100A-116-DNA-32 | Unknown | 25.48 |  |  |  |
| 65 | 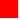 | 100A-116-DNA-33 | Unknown | 24.87 |  |  |  |
| 66 | 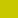 | 100A-116-DNA-33 | Unknown | 24.67 |  |  |  |
| 67 | 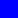 | 100A-116-DNA-34 | Unknown | 24.05 |  |  |  |
| 68 | 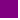 | 100A-116-DNA-34 | Unknown | 24.08 |  |  |  |
| 69 | 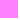 | 100A-116-DNA-35 | Unknown | 23.75 |  |  |  |
| 70 | 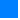 | 100A-116-DNA-35 | Unknown | 23.87 |  |  |  |
| 71 | 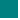 | 100A-116-DNA-36 | Unknown | 24.09 |  |  |  |
| 72 | 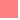 | 100A-116-DNA-36 | Unknown | 24.02 |  |  |  |

**Legend:**
NEG (NTC) - Sample cancelled due to NTC Threshold.
NEG (R. Eff) - Sample cancelled as efficiency less than reaction efficiency threshold.

| This report was generated by Rotor-Gene Q Series Software 2.3.1 (Build 49) Copyright 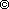2013 QIAGEN GmbH. All Rights Reserved. |
| --- |
