# Supplementary material for: Development of an in vitro diagnostic method to determine the genotypic sex of Xenopus laevis
Source: PeerJ. 2019 May 1;7:e6886. doi: 10.7717/peerj.6886 (PMC6500372; doi:10.7717/peerj.6886)
Supplement: Supplemental Information 4 — A single specimen No. 37 was analyzed in duplicate, produced Ct value for the amplification of 18S rRNA as a reference housekeeping marker. The values generated by Rotor-Gene Q real-time PCR with optimum cycling conditions, followed by 30 cycles. [file peerj-07-6886-s004.doc]

| 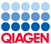 | [www.qiagen.com](http://www.qiagen.com/) |
| --- | --- |

# Quantitation Report

#### Experiment Information

| Run Name | 100A-116_DMW_Assay 2 |
| --- | --- |
| Run Start | 8/22/2018 1:42:56 PM |
| Run Finish | 8/22/2018 3:07:23 PM |
| Operator | Amin Eimanifar |
| Notes |  |
| Run On Software Version | Rotor-Gene Q Software 2.3.1.49 |
| Run Signature | The Run Signature is valid. |
| Gain Green | 5. |
| Gain Yellow | 5. |
| Machine Serial No. | 0713167 |

#### Quantitation Information

| Threshold | 0.100 |
| --- | --- |
| Left Threshold | 1.000 |
| Standard Curve Imported | No |
| Standard Curve (1) | N/A |
| Standard Curve (2) | N/A |
| Start normalising from cycle | 1 |
| Noise Slope Correction | No |
| No Template Control Threshold | % 0 |
| Reaction Efficiency Threshold | Disabled |
| Normalisation Method | Dynamic Tube Normalisation |
| Digital Filter | Light |
| Sample Page | Page 1 |
| Imported Analysis Settings |  |

#### Profile

| Cycle | Cycle Point |
| --- | --- |
| Hold 1 | Hold @ 50°C, 2min 0s |
| Hold 2 | Hold @ 95°C, 5min 0s |
| Cycling (30 repeats) | Step 1: Hold @ 95°C, 40s |
|  | Step 2: Hold @ 60°C, 60s, acquiring to Cycling A([Green][1][1],[Yellow][2][2]) |

#### Raw Data For Cycling A.Yellow


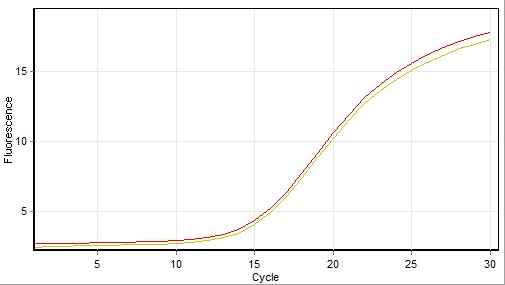


#### Quantitation data for Cycling A.Yellow


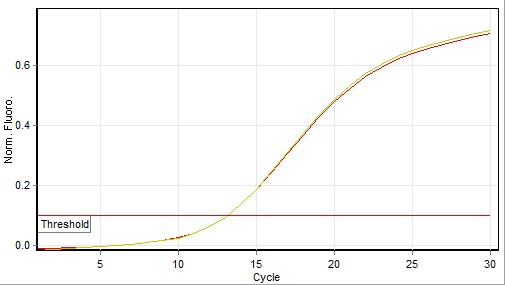


#### Standard Curve


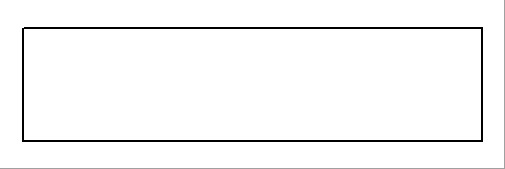


| No. | Color | Name | Type | Ct | Ct Comment | Given Conc (Copies) | Calc Conc (Copies) |
| --- | --- | --- | --- | --- | --- | --- | --- |
| 1 | 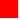 | 100A-116-DNA-37 | Unknown | 13.16 |  |  |  |
| 2 | 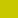 | 100A-116-DNA-37 | Unknown | 13.17 |  |  |  |

**Legend:**
NEG (NTC) - Sample cancelled due to NTC Threshold.
NEG (R. Eff) - Sample cancelled as efficiency less than reaction efficiency threshold.

| This report was generated by Rotor-Gene Q Series Software 2.3.1 (Build 49) Copyright 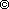2013 QIAGEN GmbH. All Rights Reserved. |
| --- |
