# Supplementary material for: Development of an in vitro diagnostic method to determine the genotypic sex of Xenopus laevis
Source: PeerJ. 2019 May 1;7:e6886. doi: 10.7717/peerj.6886 (PMC6500372; doi:10.7717/peerj.6886)
Supplement: Supplemental Information 6 — Each form was generated based on GLP guidelines and was used to record the data during histology sectioning by an experienced technician. [file peerj-07-6886-s006.pdf]

Specimen Transmittal Form

WLI Project #:	100A-116	Test Substance:		Test Organism:	*Xenopus laevis*

Treatment Group	Replicate	Animal Number	Nature of Sample*	Specimen Label	Blind ID	Comments
Uninduced	--	1	G - B	100A-116-GB-1	--	*Xenopus laevis* sample No. 1
Uninduced	--	2	G - B	100A-116-GB-2	--	*Xenopus laevis* sample No. 2
Uninduced	--	3	G - B	100A-116-GB-3	--	*Xenopus laevis* sample No. 3
Uninduced	--	4	G - B	100A-116-GB-4	--	*Xenopus laevis* sample No. 4
Uninduced	--	5	G - B	100A-116-GB-5	--	*Xenopus laevis* sample No. 5
Uninduced	--	6	G - B	100A-116-GB-6	--	*Xenopus laevis* sample No. 6
Uninduced	--	7	G - B	100A-116-GB-7	--	*Xenopus laevis* sample No. 7
Uninduced	--	8	G - B	100A-116-GB-8	--	*Xenopus laevis* sample No. 8
Uninduced	--	9	G - B	100A-116-GB-9	--	*Xenopus laevis* sample No. 9
Uninduced	--	10	G - B	100A-116-GB-10	--	*Xenopus laevis* sample No. 10
Uninduced	--	11	G - B	100A-116-GB-11	--	*Xenopus laevis* sample No. 11
Uninduced	--	12	G - B	100A-116-GB-12	--	*Xenopus laevis* sample No. 12
Uninduced	--	13	G - B	100A-116-GB-13	--	*Xenopus laevis* sample No. 13
Uninduced	--	14	G - B	100A-116-GB-14	--	*Xenopus laevis* sample No. 14
Uninduced	--	15	G - B	100A-116-GB-15	--	*Xenopus laevis* sample No. 15
Uninduced	--	16	G - B	100A-116-GB-16	--	*Xenopus laevis* sample No. 16
Uninduced	--	17	G - B	100A-116-GB-17	--	*Xenopus laevis* sample No. 17
Uninduced	--	18	G - B	100A-116-GB-18	--	*Xenopus laevis* sample No. 18
Uninduced	--	19	G - B	100A-116-GB-19	--	*Xenopus laevis* sample No. 19
Uninduced	--	20	G - B	100A-116-GB-20	--	*Xenopus laevis* sample No. 20
Uninduced	--	21	G - B	100A-116-GB-21	--	*Xenopus laevis* sample No. 21
Uninduced	--	22	G - B	100A-116-GB-22	--	*Xenopus laevis* sample No. 22
Uninduced	--	23	G - B	100A-116-GB-23	--	*Xenopus laevis* sample No. 23
Uninduced	--	24	G - B	100A-116-GB-24	--	*Xenopus laevis* sample No. 24
Uninduced	--	25	G - B	100A-116-GB-25	--	*Xenopus laevis* sample No. 25
Uninduced	--	26	G - B	100A-116-GB-26	--	*Xenopus laevis* sample No. 26
Uninduced	--	27	G - B	100A-116-GB-27	--	*Xenopus laevis* sample No. 27
Uninduced	--	28	G - B	100A-116-GB-28	--	*Xenopus laevis* sample No. 28
Uninduced	--	29	G - B	100A-116-GB-29	--	*Xenopus laevis* sample No. 29
Uninduced	--	30	G - B	100A-116-GB-30	--	*Xenopus laevis* sample No. 30
Uninduced	--	31	G - B	100A-116-GB-31	--	*Xenopus laevis* sample No. 31
Uninduced	--	32	G - B	100A-116-GB-32	--	*Xenopus laevis* sample No. 32
Uninduced	--	33	G - B	100A-116-GB-33	--	*Xenopus laevis* sample No. 33
Uninduced	--	34	G - B	100A-116-GB-34	--	*Xenopus laevis* sample No. 34
Uninduced	--	35	G - B	100A-116-GB-35	--	*Xenopus laevis* sample No. 35
Uninduced	--	36	G - B	100A-116-GB-36	--	*Xenopus laevis* sample No. 36
Uninduced	--	37	G - B	100A-116-GB-37	--	*Xenopus laevis* sample No. 37

* G = gonad, L = liver, W = whole body (organism), T = thyroid, K = kidney, R = right, E = left, B = both, Other code: ____

Transferred By (Date/Initials): **08.15.2018/AE** Comments: Emmanuel far. H

Condition upon receipt: Good

Received By (Date/ Initials): MK 8.22.18

EAG LABORATORIES

Study Specific Specimen Processing Instructions

Project Number: 100A-116

Type of tissues (e.g. gonad, liver, whole body) to be processed:<br>Gonads of each adult frog	
List specimens to be processed: All frogs from this study that are submitted to histology lab are to be sectioned and mounted.	Preservative for Specimen<br>10% Formalin solution **X** N/A ☐<br>Other (Describe in comment) ☐
Preparatory sectioning (depth of first section)<br>Coarse face until organ of interest is first observed **X**<br>Coarse face to midline of organ ☐<br>Other (Describe in comment)	*Gross dissection instructions*<br>Remove head and tail ☐<br>Other (Describe in Comments) **X** N/A ☐
Orientation of tissue/organ within block (for specified sectioning method):<br>Longitudinal (dorsum nearest to block face) **X**<br>Longitudinal (side of body nearest block face) ☐<br>Transverse ☐ Other (Describe in comment) ☐	*Minimum number of Levels per Specimen*<br>Three **X** Five ☐ Other ______
Number of serial sections per level:<br>One **X** Two ☐ Other (Describe in comment) ☐	Number of sections per slide:<br>One ☐ Two ☐ Three **X** Other (Describe in comment) ☐
Preliminary estimate of distance between levels:<br>30 microns ☐ 50 microns **X** Other (Describe in comment)	Section thickness<br>4 – 5 microns **X** Other ______
Comment/Special Instructions[a]:	

[a] Special instructions should include: Relevant instructions protocol not given above or related to presence of grossly visible lesions or conditions. List any specimens that are to be processed to a certain point and then held prior to sectioning.

Initials: AE **Date:** 08-15-2018

EAG - Easton

Histology Processing Log

Project Number:	100A-116

Specimen ID[a]	Gross Dissection[d] (Init/Date)	Cassette ID (*)	Placed in VIP[b] (Init/Date/Progr)[c]	Removed from VIP (Init/Date)	Embedded[d] (Init/Date)	Sectioning (Init/Date/Meth[bd])	Slide Numbers	Stained[bd] (Init/Date/Meth)	Cover-slipped[bd] (Init/Date/Meth)
1	N/A		MK / 8-28-18 / 02	MK / 8-29-18	MK / 8-29-18	MK / 9-5-18 / SS	S1-2	MK / 9-21-18 / SS	MK / 9-21-18 / SS
2									
3									
4									
5						MK / 9-5-18 / SS	S1-2		
6						MK / 9-6-18 / SS	S1-2		
7									
8									
9									
10						MK / 9-6-18 / SS	S1-2		
11						MK / 9-7-18 / SS	S1-2		
12									
13									
14									
15						MK / 9-7-18 / SS	S1-2		
16									
17									
18									
19						MK / 9-7-18 / SS	S1-2		
20						MK / 9-10-18 / SS	S1-2		
21	N/A		MK / 8-28-18 / 02	MK / 8-29-18	MK / 8-29-18	MK / 9-10-18 / SS	S1-3	MK / 9-21-18 / SS	MK / 9-21-18 / SS

Notes:
(*) Same as specimen ID @ MK 8-27-18

[a] Specimen ID is the content of this column appended to the Project Number.
[b] Instrument identifications are as follow unless otherwise noted:
Tissue Processor: HTP#1, Microtome: HMT#1, Autostainer: HLA#1, Coverslipper: HCS#1
[c] Initials indicate the person performing the task. Date is the date of completion of the task. Program (Prog) codes are S0=Small organ, WA=Whole Animal
[d] Method (Meth) code SS indicates that the sectioning was performed according to the study specific method from the form "Study Specific Specimen Processing Instructions" submitted to the histology lab with the specimens. Method = OT indicates that special sectioning procedures were used. Describe in notes section..

EAG - Easton

Histology Processing Log

Project Number:	100A-114

Specimen ID[a]	Gross Dissection[d] (Init/Date)	Cassette ID ④	Placed in VIP[b] (Init/Date/Progr)[c]	Removed from VIP (Init/Date)	Embedded[d] (Init/Date)	Sectioning (Init/Date/Meth[bd])	Slide Numbers	Stained[bd] (Init/Date/Meth)	Cover-slipped[bd] (Init/Date/Meth)
22	N/A		MC / 8-28-18 / 02	MC / 8-29-18	MC / 8-29-18	MC / 9-10-18 / SS	S1-3	MC / 9-21-18 / SS	MC / 9-21-18 / SS
23						↓	↓		
24						MC / 9-10-18 / SS	S1-3		
25						MC / 9-11-18 / SS	S1-~~3~~2 ①		
26									
27									
28						↓	↓		
29						MC / 9-11-18 / SS	S1-3		
30						MC / 9-17-18 / SS	S1-3		
31									
32									
33						↓	↓		
34						MC / 9-17-18 / SS	S1-3		
35						MC 9-18-18 / SS	S1-3		
36			↓			↓	↓	↓	↓
37	N/A		MC / 8-28-18 / 02	MC / 8-29-18	MC / 8-29-18	MC / 9/18/18 / SS	S1-3	MC / 9-21-18 / SS	MC / 9-21-18 / SS

Notes:

④ Same as specimen ID @ MC 8-27-18

① @ MC 9-11-18

[a] Specimen ID is the content of this column appended to the Project Number.

[b] Instrument identifications are as follow unless otherwise noted:
Tissue Processor: HTP#1, Microtome: HMT#1, Autostainer: HLA#1, Coverslipper: HCS#1

[c] Initials indicate the person performing the task. Date is the date of completion of the task. Program (Prog) codes are S0=Small organ, WA=Whole Animal

[d] Method (Meth) code SS indicates that the sectioning was performed according to the study specific method from the form "Study Specific Specimen Processing Instructions" submitted to the histology lab with the specimens. Method = OT indicates that special sectioning procedures were used. Describe in notes section..

Appendix A-Hematoxylin and Eosin Staining and Reagent Rotation Scheme

Step	Reagent	Time (Minutes)[a]	Rotation Scheme
1	Everclear	2:00	Discard & replace with 2
2	Everclear	1:00	Rotate to 1 & replace with 3
3	Everclear	1:00	Rotate to 2 & replace with new Everclear
4	100% ETOH	1:00	Replace
5	95% ETOH	1:00	Replace
6	Wash in Tap Water	1:00	N/A
7	Hematoxylin 1	1:00	Discard 200 -300 mL & replace with 200-300 mLfrom 8
8	Hematoxylin 2	1:00	Replenish with 200-300 mL new Hematoxylin
9	Wash in Tap Water	1:00	N/A
10	Clarifier	1:00	Replace
11	Wash in Tap Water	1:00	N/A
12	Bluing Reagent	1:00	Replace
13	Wash in Tap Water	1:00	N/A
14	95% ETOH	1:00	Replace
15	Eosin Y Alcoholic	1:00	Discard 200 -300 mL & replace with 200-300 mL new Eosin Y Alcoholic
16	95% ETOH	0:30	Replace
17	100% ETOH	1:00	Replace
18	100% ETOH	1:00	Replace
19	100% ETOH	1:00	Replace
20	Everclear	2:00	Replace with 21
21	Everclear	1:00	Rotate to 20 & replace with new Everclear
	Xylene	at least 2:00	Replace as needed

[a] Steps 1-21 gentle agitation is performed during the immersion period.

CERTIFIED COPY
OF THE ORIGINAL
INITIALS DATE
10-1-14

EAG LABORATORIES

Transmittal of Slides, Blocks and Preserved Remnants to Temporary Storage

EAG-Easton Project No. #:	100A - 116	Sponsor Project # (If Applicable)	

Check If Included	Number of Folders	Slides Included (List)	Condition on Receipt[c] (Completed by Archivist)
☑ Slides	1	100A-116-GB-1-S1 ⟶ 100A-116-GB-37-S3	

Check If Included	Number of Boxes	Blocks Included (List)	Condition on Receipt[c] (Completed by Archivist)
☑ Blocks	1	100A-116-GB-1 ⟶ 100A-116-GB-37.	

Check If Included	Number of Boxes	Specimens Included (List)	Condition on Receipt[c] (Completed by Archivist)
☐ Preserved remnants			

[c] I=intact; L-leaking,; B=broken, samples not in container; 0=other (explanation required)

Notes:

Total blocks - 37 blocks
Total Slides - 90 slides

Transferred by (Signature/Date):

[signature] 1-7-19

Received by (Signature/Date:

[signature] 1-7-19
