# Supplementary figures and images for: Development of an in vitro diagnostic method to determine the genotypic sex of Xenopus laevis
Source: PeerJ. 2019 May 1;7:e6886. doi: 10.7717/peerj.6886 (PMC6500372; doi:10.7717/peerj.6886)

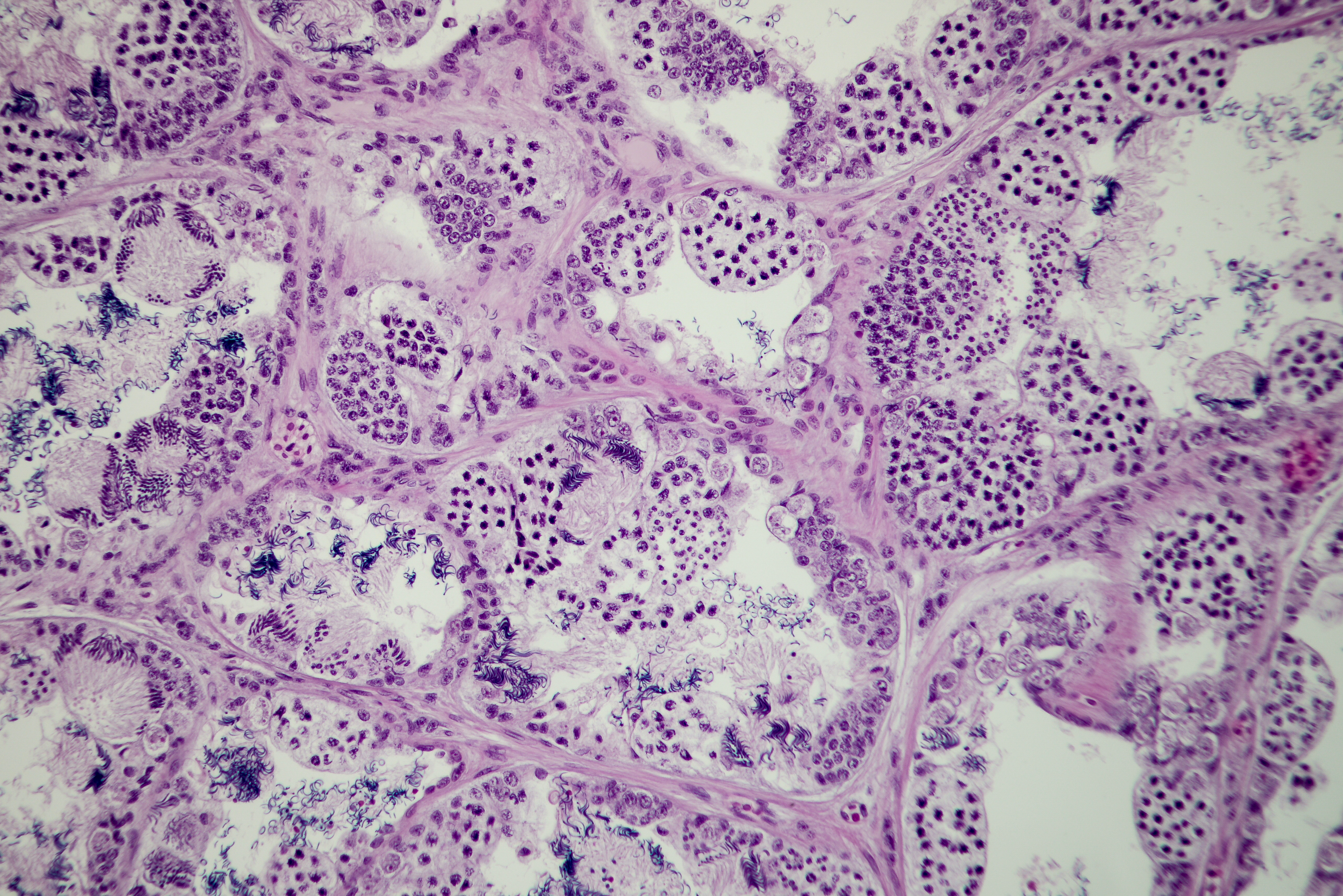

Supplement: Supplemental Information 7 — The image produced by the histological sectioning of the specimen No. 5 following the procedures published in OECD-LAGDA guideline, stained with HE. A male biological endpoint was confirmed by a certified pathologist. [file peerj-07-6886-s007.jpg]

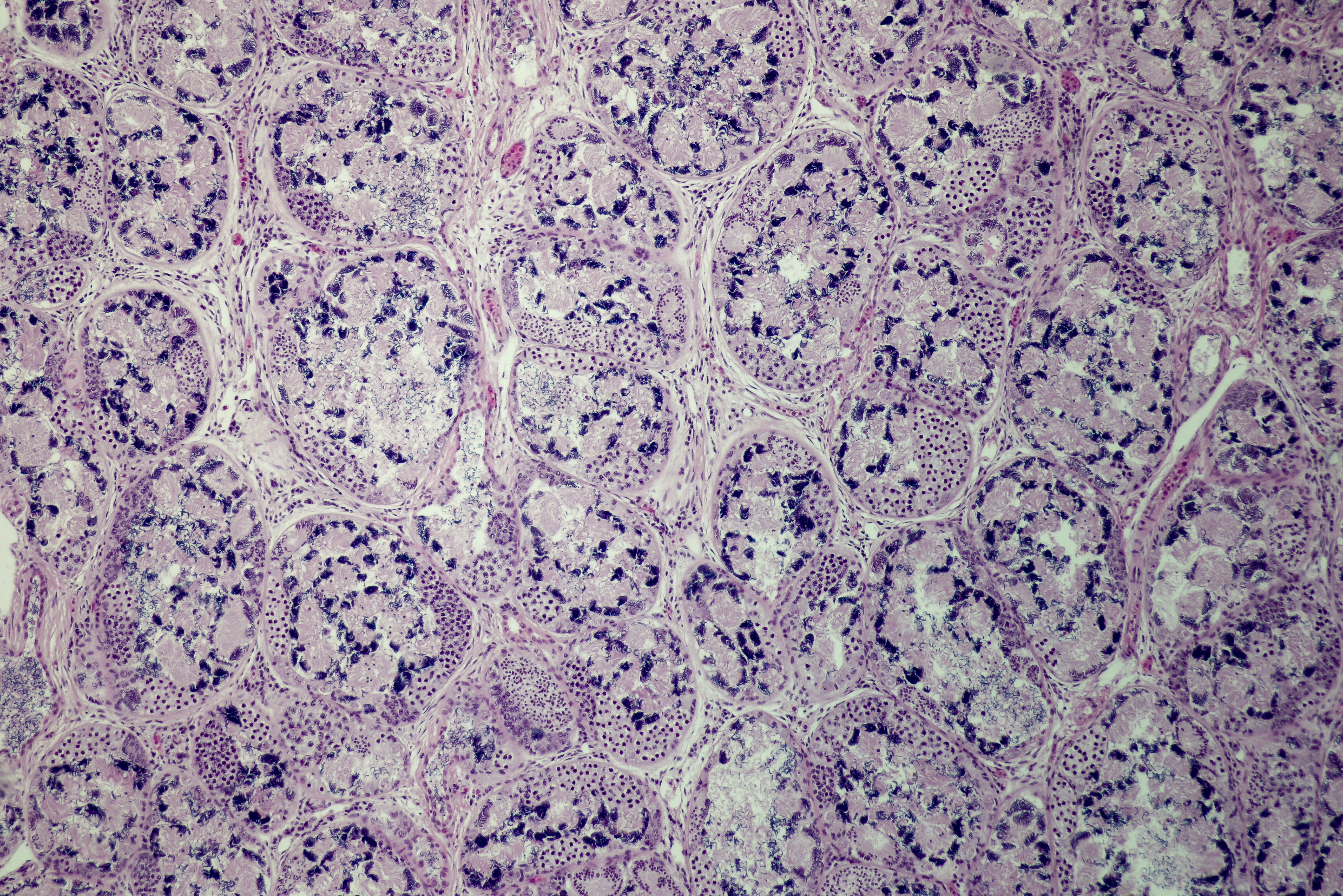

Supplement: Supplemental Information 8 — The image produced by the histological sectioning of the specimen No. 2 following the procedures published in OECD-LAGDA guideline, stained with HE). A male biological endpoint was confirmed by a certified pathologist. [file peerj-07-6886-s008.jpg]
